# Functional Plasticity of the Visual System in Multiple Sclerosis

**DOI:** 10.3389/fneur.2015.00079

**Published:** 2015-04-08

**Authors:** Antonio Gallo, Alvino Bisecco, Simona Bonavita, Gioacchino Tedeschi

**Affiliations:** ^1^Department of Medical, Surgical, Neurological, Metabolic and Aging Sciences, Second University of Naples, Naples, Italy; ^2^MRI Center SUN-FISM, Neurological Institute for Diagnosis and Care “Hermitage Capodimonte”, Naples, Italy

**Keywords:** multiple sclerosis, visual system, plasticity, MRI, functional

Multiple sclerosis (MS) can affect the visual system at all anatomical sites from the retinal nerve fiber layer (RNFL) to the visual cortex. MS demyelinating lesions most commonly occur at the level of the optic nerve, causing acute episodes of optic neuritis (ON) (1). ON usually determines a moderate to severe visual impairment, followed by a complete or near complete clinical recovery within a few weeks. However, even after visual recovery, a permanent structural and functional damage of the anterior visual system can be detected by visual evoked potentials (VEP), MRI, and RNFL imaging (2). Two hypotheses, which do not exclude each other, are commonly advocated to explain such clinical–paraclinical paradox. The first suggests that axons of the optic nerve can maintain a normal clinical function up to a critical threshold of nerve fibers loss, as a result of the intrinsic structural reserve (also known as neuroaxonal redundancy) (3). The second supports the role of adaptive functional changes taking place at the level of striate and extra-striate visual cortical areas (4–8).

While the first hypothesis is extremely difficult to test *in vivo* – even applying advanced structural MRI techniques such as diffusion tensor imaging (DTI) – the second hypothesis has been repeatedly tested by functional MRI (fMRI) during visual stimuli or, more recently, resting conditions.

Adaptive neuroplasticity is formally defined as the reorganization of distributed patterns of brain activity that accompany action, perception, and cognition, and that compensate for impaired function resulting from disease or brain injury (9). On the other hand, non-adaptive neuroplasticity refers to functional cortical reorganization not associated with any benefit on brain function.

Using visual-stimulated fMRI, MS patients in the early stages of ON have consistently shown a reduced response to visual stimuli in the primary visual (striate) cortex when compared to healthy controls (HCs) (4–6). These investigations support the hypothesis that during the acute phase of ON, the function of the primary visual cortex is significantly affected by a substantial reduction of the afferent inputs due to damage to the optic nerve. Other fMRI studies on ON have widened the view has led to wider in depth knowledge reporting significant functional changes occurring at the level of peristriate and extra-striate visual cortices as well (6–8, 10). Recruitment of extra-striate areas, in particular, has been shown at the level of the insula, claustrum, thalami, lateral geniculate nuclei, corpus striatum, orbitofrontal and lateral temporal cortices, posterior parietal cortex, and lateral occipital complexes (LOCs) (7, 9, 10). Among the abovementioned areas – for many of which the exact effect (i.e., adaptive or non-adaptive) on visual recovery still needs to be clarified – LOCs, higher order visual areas located within the ventral processing stream and involved in identification and recognition of objects, have consistently showed relevant functional changes associated with ON (7, 8). In a preliminary study conducted during the earliest stages of ON, patients with a better visual acuity at baseline showed a stronger activation of the LOCs, independently of VEP latencies and severity of optic nerve damage (7). The potential adaptive role of LOCs on visual recovery after ON was further supported by a longitudinal study, showing that over-activation of LOCs at baseline was associated with a better visual outcome at 1 year, independently of anterior visual pathway structural damage. Such findings were strengthened by the negative prognostic value of reduced LOCs fMRI responses at baseline (8).

Although visual-stimulated fMRI studies have allowed to comprehend relevant aspects of functional changes occurring in visual cortical areas during ON, they are intrinsically limited by the fact that the measured signal changes mostly reflect the degree of anterior visual pathway damage rather than intrinsic activity of visual cortical areas. Moreover, since optic nerve damage is highly variable between subjects with ON, there is a wide inter-subject variability, which further undermines the results and interpretation of visual-stimulated fMRI studies.

Given these premises, resting-state (RS) fMRI has a relevant role as it overcomes most of the abovementioned limitations, and allows to explore spontaneous (self-generated) activity of cortical visual areas.

In the study by Roosendaal et al., – the first to explore the visual RS network (V-RSN) together with other major RSNs – the authors did not find any significant changes in both clinical isolated syndrome (CIS) and relapsing remitting (RR) MS patients when compared to each other and HCs. Such findings were not surprising since the study was not designed to explore the relationship between V-RSN changes and ON. Indeed, no historical data on previous ON, no clinical/instrumental evaluation of the visual function, and no measures of optic nerve damage were acquired (11).

Using a similar approach, Faivre et al. explored all main RSNs, including the V-RSN, to compare early RRMS patients with HCs. Similarly to the Roosendaal study, data acquisition and analysis did not take into account previous episodes of ON. Nevertheless, the authors were able to demonstrate functional rearrangements inside multiple regions of the V-RSN (i.e., lingual gyrus, left middle occipital gyrus, and left cuneus), thus showing significant neuroplastic changes of this RSN in MS patients (12).

In order to specifically explore the effect of previous ON on intrinsic V-RSN connectivity, we investigated a population of 30 RRMS patients [16 without (nON-MS) and 14 with (ON-MS) previous ON] and 15 HCs (13). For this purpose, all subjects underwent a 3TMRI including RS-fMRI data acquisition, a neurological examination, and a thorough ophthalmologic evaluation, based on the assessment of visual acuity as well as the measurement of RNFL thickness. When the entire group of RRMS patients was compared to HCs, a weakened V-RSN connectivity was found in RRMS patients at the level of inferior peristriate cortices (along the fusiform gyri), bilaterally. The subsequent comparison of ON-MS versus nON-MS patients showed a spot of stronger functional connectivity (FC) in the right extra-striate cortex (along the middle occipital gyrus; MOG) as well as a spot of reduced FC in the right inferior peristriate cortex, in ON-MS patients. Notably, all detected V-RSN changes did not co-localize with regional gray matter atrophy.

Since our patients with RRMS showed a significant damage of the anterior visual pathways and until now there have been no other RS studies conducted on ON, we compared our results with those obtained in non-MS diseases affecting the anterior visual pathways, such as Leber hereditary optic neuropathy and early blindness (14, 15). In agreement with our findings, these studies reported significant functional changes at the level of the V-RSN (14, 15).

Our data, therefore, confirm and complement previous photic-stimulated fMRI studies, showing that visual recovery after ON might be associated with cortical reorganization within extra-striate visual areas (6, 7, 10). In particular, one might speculate that an enhanced RS FC at the level of the right MOG might either reflect a sustained and protracted recruitment or a loss of inhibition of this area after ON.

Although the study of the V-RSN has certainly added relevant information on ON-related neuroplastic changes taking place at the level of the striate and extra-striate cortex, the current lack of longitudinal studies do not allow to discriminate between adaptive ad non-adaptive changes.

## Conclusion

To date, visual plasticity of the visual system in MS has been investigated especially after ON.

Relevant cortical functional changes have been consistently described at the level of striate and extra-striate areas using either photic-stimulated or RS-fMRI studies.

Available evidences also suggest that some of the observed neuroplastic changes, in particular, those occurring at the level of the occipital extra-striate cortex (e.g., LOCs and MOG), might act as adaptive neuroplasticity, thus impacting positively on visual recovery after ON.

Once the functional plasticity of the visual system related to ON in MS will be further elucidated it might become a useful model to evaluate the effect of new neuroprotective or (adaptive) plasticity-promoting molecules.

## Perspectives

Future investigations will have to further assess adaptive and non-adaptive neuroplasticity of the visual system in MS patients. More specifically, cross-sectional and longitudinal studies will have to address, which cortical functional changes are associated to functional recovery after ON. Once the methodology will be set up and standardized in a multicenter context will be able to assess the effect of old or new MS drugs on mechanisms of visual recovery after ON.

## Conflict of Interest Statement

The authors declare that the research was conducted in the absence of any commercial or financial relationships that could be construed as a potential conflict of interest.
